# Epidemiology of patients with asthma in Korea: Analysis of the NHISS database 2006–2015

**DOI:** 10.1016/j.waojou.2023.100768

**Published:** 2023-04-19

**Authors:** Jung-Hyun Kim, Hyesung Lee, So-Young Park, Ju-Young Kim, Sun Hee Choi, Hyouk-Soo Kwon, Woo-Jung Song, Sae-Hoon Kim, Jinho Yu, Dae Jin Song, You Sook Cho, Dae Hyun Lim, Young-Joo Cho, Suk-Il Chang, Tae-Bum Kim

**Affiliations:** a Department of Allergy and Clinical Immunology, Asan Medical Center, University of Ulsan College of Medicine, Seoul, South Korea; b Department of Internal Medicine, Korean Armed Forces Capital Hospital, Seongnam, South Korea; c Department of Biohealth Regulatory Science School of Pharmacy, SungKyunKwan University, Seongnam, South Korea; d Department of Internal Medicine, Chung-Ang University College of Medicine, Seoul, South Korea; e Division of Pulmonary, Allergy and Critical Care Medicine, Chung-Ang University Gwangmyeong Medical Center, Gwangmyeong, South Korea; f Department of Internal Medicine, Dankook University Hospital, Cheonan, South Korea; g Kyung Hee University School of Medicine, Seoul, South Korea; h Department of Internal Medicine, Seoul National University Bundang Hospital, Seongnam, South Korea; i Department of Pediatrics, Asan Medical Center, University of Ulsan College of Medicine, Seoul, South Korea; j Department of Pediatrics, Korea University College of Medicine and Environmental Health Center for Childhood Asthma, Korea University Anam Hospital, Seoul, South Korea; k Department of Pediatrics, School of Medicine, Inha University, Incheon, South Korea; l Department of Internal Medicine, Ewha Women University School of Medicine, Seoul, South Korea; m Department of Internal Medicine, Sung-Ae Hospital, Seoul, South Korea

**Keywords:** Asthma, Claims data, Severity

## Abstract

**Background:**

There has been a concerning increase in the prevalence and socioeconomic burden of asthma in Korea. Korea's National Health Insurance System (NHIS) covers insurance payment and claims management for all Koreans. Using National Health Insurance Sharing Service (NHISS) claims data. This study aimed to investigate patterns of healthcare utilization and direct cost in patients with asthma over a 10-year period.

**Methods:**

In this retrospective population-based study, we examined NHISS claims records between July 2005 and June 2016 and investigated healthcare utilization among patients with asthma based on age group and severity of disease (non-severe asthma [NSA] and severe asthma [SA]).

**Results:**

From 2006 to 2015, the total number of patients with asthma in Korea steadily increased from 743 968 to 2 286 309, with a corresponding increase in prevalence from 1.62% to 4.74%. The proportion of patients with SA decreased from 3.16% in 2006 to 1.56% in 2015; the proportion was consistently higher in men than in women. In addition, patients with SA had a higher cost per outpatient visit than patients with NSA, and the number of outpatient visits per year increased. The inhaled corticosteroid (ICS) prescription rate among patients with asthma decreased from 22.9% in 2006 to 15.7% in 2015. Furthermore, for a period of 10 years, more than 40% of patients with SA have been prescribed short-acting β-2 agonists (SABAs).

**Conclusions:**

Although patients with SA comprised a small proportion of patients with asthma, they incurred greater medical costs per person. The pharmaceutical prescription pattern indicated a lack of ICS-based prescriptions and frequent SABA prescriptions.

## Introduction

Asthma is one of the most common chronic airway diseases, affecting approximately 300 million people worldwide.^1^ The prevalence of asthma varies from 0.2 to 21.0% in adults and 2.8–37.6% in children.^2^^,^^3^ Studies have reported that the prevalence of asthma in Korea ranges from 1.6 to 4.1% in adults and 5.7–28.8% in children, which has been gradually increasing annually.^4^, ^5^, ^6^, ^7^ The high prevalence of asthma results in a high burden on medical resources.

Korea has a unique National Health Insurance System (NHIS) in which the governmental insurer has been covering insurance payments and claims management for the country's population since 1989. Koreans using the medical system (private or public) in Korea must be enrolled in the NHIS. All claims data for healthcare utilization are assessed by the system according to the relevance of the claim, and the government subsequently subsidizes the cost. If a prescription is evaluated as inadequate for a diagnosis by the NHIS, it is deleted from the claims data, and the medical institution pays the cost. Therefore, claims data in Korea usually contain generally accepted prescribed tests and treatments for the corresponding disease, serving as an accurate reflection of real clinical practice.^8^^,^^9^

In this study, to create evidence-based data for future asthma-related healthcare policies and to identify the economic burden associated with asthma severity, we investigated healthcare utilization patterns and direct costs in patients with asthma over a 10-year period using National Health Insurance Sharing Service (NHISS) claims data. We also determined the differences in healthcare utilization and costs depending on age group and disease severity.

## Methods

### Data sources

We analyzed NHISS claims data from July 2005 to June 2016 extracted from every hospital (this service covers all citizens in this country). The data are in accordance with the eighth, ninth, and tenth revisions of the International Statistical Classification of Diseases and Related Health Problems (ICD-8, 9, 10). The data included demographic information about each patient, details of prescribed drugs, prescribed medical tests or procedures, and healthcare expenditure accepted by the NHIS. We extracted the medical information of patients with asthma from the NHISS database; this was a modification of our previous study.^4^^,^^8^, ^9^, ^10^ Patients who met the following criteria were included: 1) age >5 years; 2) two or more claims in 1 year with ICD codes (J45.x–J46.x) for the principal or 4 additional diagnoses of asthma, and prescription of at least 1 asthma-related medication 6 months before and 6 months after the first claim of the year. Asthma-related medications included inhaled corticosteroids (ICSs), long-acting β-2 agonists (LABAs), ICSs and LABAs combined in a single inhaler (ICSs/LABAs), oral leukotriene antagonists (LTRAs), short-acting β-2 agonists (SABAs), long-acting muscarinic antagonists (LAMAs), short-acting muscarinic antagonists (SAMAs), systemic β agonists, and theophylline derivatives.^8^, ^9^, ^10^ The codes for treatment medications, tests, and procedures were collected based on all publicly available data published by the NHISS between 2005 and 2016.

### Study population and working definitions

Patients were categorized according to the severity of their asthma. Patients who were prescribed canisters of high-dose ICSs for >6 months of the year and who were prescribed canisters of low-dose ICSs for more than 12 months because of asthma-related claims were considered to have severe asthma (SA). All other patients were considered to have non-severe asthma (NSA). Regarding patients with SA, we could not apply the latest SA definition of the Global Initiative for Asthma (GINA) guidelines, which is “asthma that is uncontrolled despite adherence with optimized high dose ICS-LABA therapy and treatment of contributory factors, or that worsens when high dose treatment is decreased.” Instead, we focused on the use of continuous high-dose ICSs per the SA definition (Table 1). Direct medical costs were defined as the sum of claims charges covered by the NHIS, including hospitalization costs, outpatient services, and outpatient medication costs. Prevalence was age-standardized (using midyear residents as the reference population) and reported by year. All statistical analyses were performed using SAS 9.2 software (SAS Institute, Cary, NC, USA).

**Table 1:** Operational definitions for asthma and related outcomes from the claims database.

	Operational definition of diagnosis
Asthma-related claim	Claim with ICD-8, 9, and 10 codes (J45.x–J46.x) for the principal or four additional diagnoses^a^ of asthma and at least one asthma-related medication^b^.
Hospital admission due to asthma exacerbation	Asthma-related claim with systemic corticosteroid prescription for ≥1 days and nebulizer treatment^c^.
Emergency visit due to asthma exacerbation	Asthma-related claim plus ER code with systemic corticosteroid prescription for ≥1 days and nebulizer treatment.
Outpatient clinic visit due to asthma exacerbation	Asthma-related claim with systemic steroid prescription for 2 or more days.
Patients with	
Asthma	At least 2 asthma-related claims occurred between 6 months before and 6 months after the first claim data of the year.
Severe asthma^d^	Patients who were prescribed with a canister of inhaled corticosteroids (ICSs) equivalent to a canister of high-dose ICSs for >6 months of the year with asthma-related claims. (They must simultaneously satisfy the following criterion: prescribed ICS canisters equivalent to greater than the sum of 12 months prescription of low-dose ICS.)
Non-severe asthma	All other patients.

∗All patients are >5 years of age.

a Principal or 4 additional diagnoses: The ICD diagnosis codes that apply to the prescription. In the case of prescriptions for which 5 or more diagnoses are entered, the principal diagnosis code and up to 4 additional diagnosis codes in the following order are applied to the analysis.

b Asthma-related medication: inhaled corticosteroids (ICSs), long-acting β-2 agonists (LABAs), ICSs and LABAs combined in a single inhaler (ICSs/LABAs), oral leukotriene antagonists, short-acting β-2 agonists (SABAs), long-acting muscarinic antagonists (LAMAs), short-acting muscarinic antagonists (SAMAs), systemic β agonists, and theophylline derivatives.

c Nebulizer treatment: code for nebulizer treatment or nebulizer medications (ICSs, SABAs, and ipratropium bromide).

d Severe asthma: we did not apply the definition of severe asthma in the GINA guidelines (2023); asthma that is uncontrolled despite adherence to optimized high-dose ICS-LABA therapy and treatment of contributory factors or that worsens when high-dose treatment is decreased

## Results

### Prevalence of asthma

**Fig. 1:**
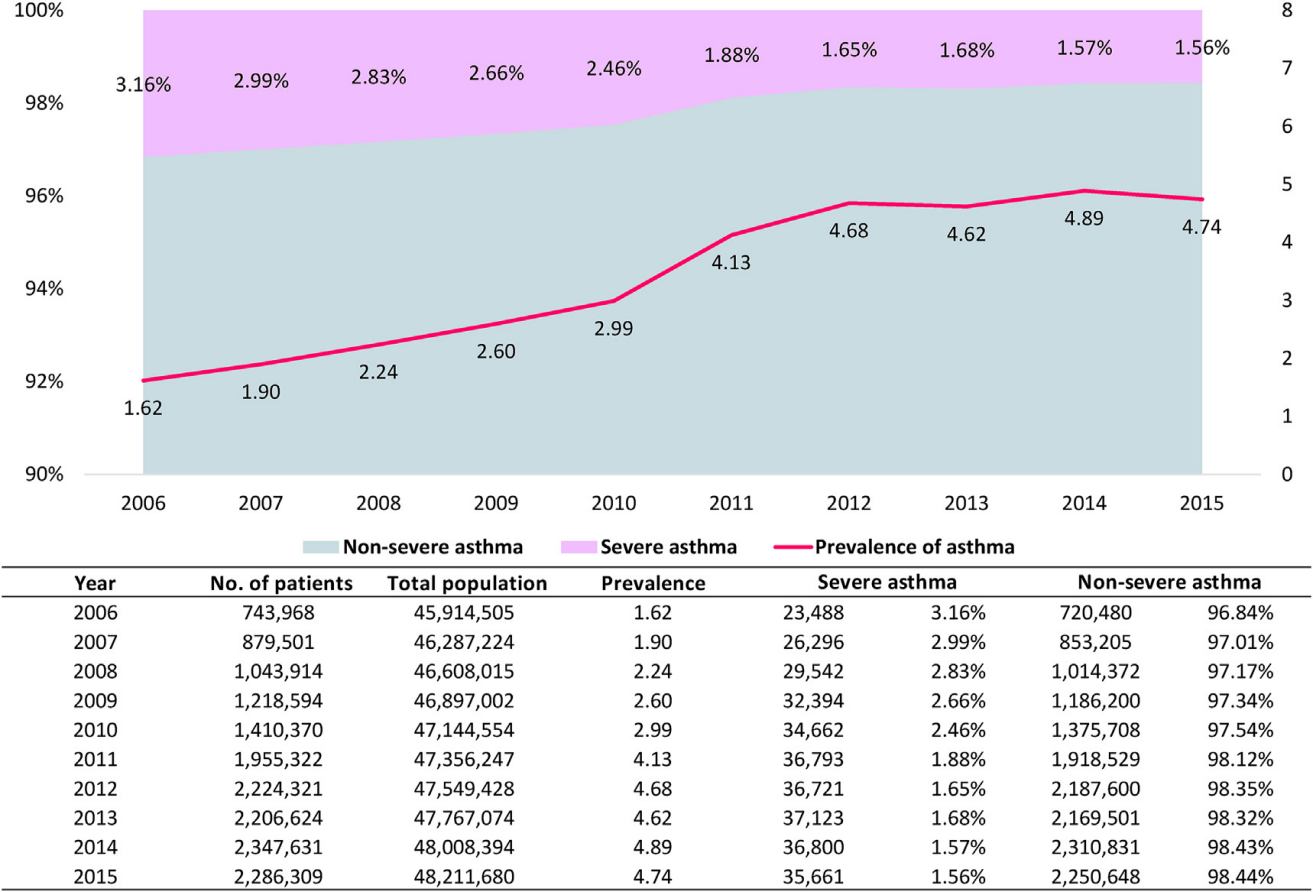
Asthma severity and prevalence by year

The proportion of patients with asthma steadily increased from 743 968 in 2006 to 2 286 309 in 2015, and asthma affected a greater number of women compared with men throughout this period. The prevalence of asthma in Korea gradually increased from 1.62% in 2006 to 4.74% in 2015 (Fig. 1). The prevalence of asthma among children younger than 18 years was 1.58% in 2006 and increased to 8.65% in 2015. The highest prevalence was observed in adults aged 60 years or older (Table E1). The proportion of patients with SA among all patients with asthma gradually decreased from 3.16% in 2006 to 1.56% in 2015 (Fig. 1). In addition, the prevalence of asthma among women was consistently higher than that among men over the entire 10-year period. When each group by sex was divided into age groups, the prevalence of asthma in children younger than 18 years and patients older than 80 years remained higher in women than in men (Table E1). However, the proportion of patients with SA was consistently higher in men than in women (Figure E1).

### Healthcare utilization in patients with asthma

Over 10 years, the proportion of patients with asthma who experienced hospitalization in a year remained relatively constant at 1.9–2.2%. The proportion of patients with a history of asthma-related emergency room visits decreased gradually from 2.4% in 2006 to 2.0% in 2015, whereas asthma-related intensive care unit (ICU) admissions remained at 0.3–0.5% per year over the entire 10-year period. Outpatient visits averaged 4.49 visits per patient in 2006 and increased to an average of 6.50 visits in 2008; however, a decrease was observed thereafter to 5.18 visits in 2016. The cost per visit and annual resource costs peaked in 2006 and decreased thereafter (Table 2).

**Table 2:** Healthcare utilization by patients with asthma and other asthma-related healthcare services for a period of 10 years.

	2006		2007		2008		2009		2010		2011		2012		2013		2014		2015	
Total	743 968		879 501		1 043 914		1 218 594		1 410 370		1 955 322		2 224 321		2 206 624		2 347 631		2 286 309	
Hospitalization																				
Yes (n, %)	16 536	(2.2)	19 120	(2.2)	22 678	(2.2)	25 559	(2.1)	29 807	(2.1)	39 963	(2.0)	45 224	(2.0)	41 915	(1.9)	45 083	(1.9)	49 875	(2.2)
Number of hospitalizations^a^	1.52		1.53		1.61		1.62		1.70		1.68		1.67		1.69		1.66		1.59	
Cost per year^b^	2242		2645		2493		2328		2905		3603		3405		3576		3603		3331	
Cost per admission^c^	1471		1726		1552		1434		1707		2147		2032		2112		2167		2093	
Hospitalization days																				
Per year (mean)	18.79		19.66		21.14		21.65		23.65		25.41		24.77		25.12		23.83		21.89	
Per admission (mean)	12.35		12.85		13.18		13.37		13.92		15.16		14.81		14.87		14.38		13.81	
ER visit																				
Yes (n, %)	18 114	(2.4)	21 025	(2.4)	24 335	(2.3)	29 488	(2.4)	33 794	(2.4)	43 642	(2.2)	47 967	(2.2)	43 815	(2.0)	45 324	(1.9)	46 612	(2.0)
Number of ER visits	1.26		1.26		1.27		1.27		1.28		1.27		1.29		1.29		1.30		1.28	
Cost per year^b^	1307		1528		1410		1263		1538		1986		1897		1998		2034		2016	
Cost per visit^c^	1022		1198		1096		983		1177		1544		1452		1533		1547		1550	
ICU admission																				
Yes (n, %)	2597	(0.3)	2951	(0.3)	3564	(0.3)	4208	(0.3)	5377	(0.4)	9158	(0.5)	9464	(0.4)	8709	(0.4)	8452	(0.4)	8591	(0.4)
Number of ICU admissions^a^	1.09		1.09		1.10		1.12		1.14		1.22		1.22		1.22		1.21		1.23	
Cost per year^b^	4526		5161		4884		4570		5662		7063		6887		7186		7556		7673	
Cost per admission^c^	4145		4709		4423		4091		4981		5798		5649		5874		6244		6257	
Outpatient visit																				
Number of outpatient visits^a^	4.49		5.70		6.50		6.42		6.17		5.40		5.46		5.40		5.29		5.18	
Cost per year^b^	190		196		165		145		160		137		113		112		112		109	
Cost per visit^b^	44		37		27		24		28		27		22		22		23		22	

∗All costs are presented in USD as the average annual exchange rate for each year (1 USD = 955.74 Korean won [KRW] in 2006, 1 USD = 929.20 KRW in 2007, 1 USD = 1101.93 KRW in 2008, 1 USD = 1276.18 KRW in 2009, 1 USD = 1156.06 KRW in 2010, 1 USD = 1108.09 KRW in 2011, 1 USD = 1126.43 KRW in 2012, 1 USD = 1094.97 KRW in 2013, 1 USD = 1053.30 KRW in 2014, and 1 USD = 1132.10 KRW in 2015).

a Number of admissions to the hospital or hospital visits per person per year.

b Direct medical costs per person per year.

c Direct medical costs per person per admission or hospital visit.

Most adult patients with asthma received medical treatment through the department of internal medicine, and in the case of otolaryngology, there was an even distribution of patients with asthma across all age groups. However, more than 90% of pediatric patients were <17 years old (Table E2).

### Medical costs for patients with asthma

Increasing admission costs resulted in a gradual increase in the medical cost of hospitalization per patient per year. Although the outpatient cost per person per year and cost per visit per person decreased in 2015 compared with that in 2006, the annual hospitalization cost per patient per year and cost per admission increased by approximately 48% in 2015 compared with that in 2006 (2242 USD to 3331 USD; Table 2). Similarly, the cost of emergency room visits and ICU admission increased by more than 50% in terms of the cost per person and cost per visit in 2015 compared with that in 2006 (4526 USD to 7623 USD; Table 2).

### Healthcare use and direct costs according to the severity of asthma

**Fig. 2:**
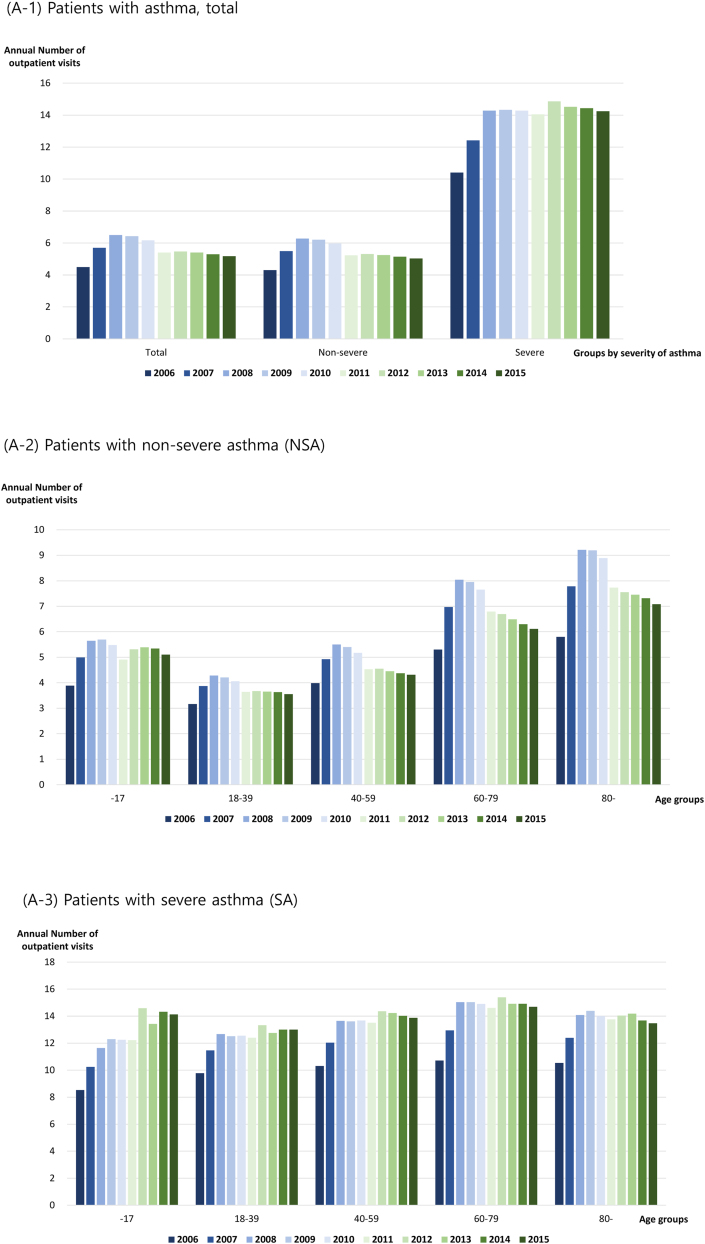

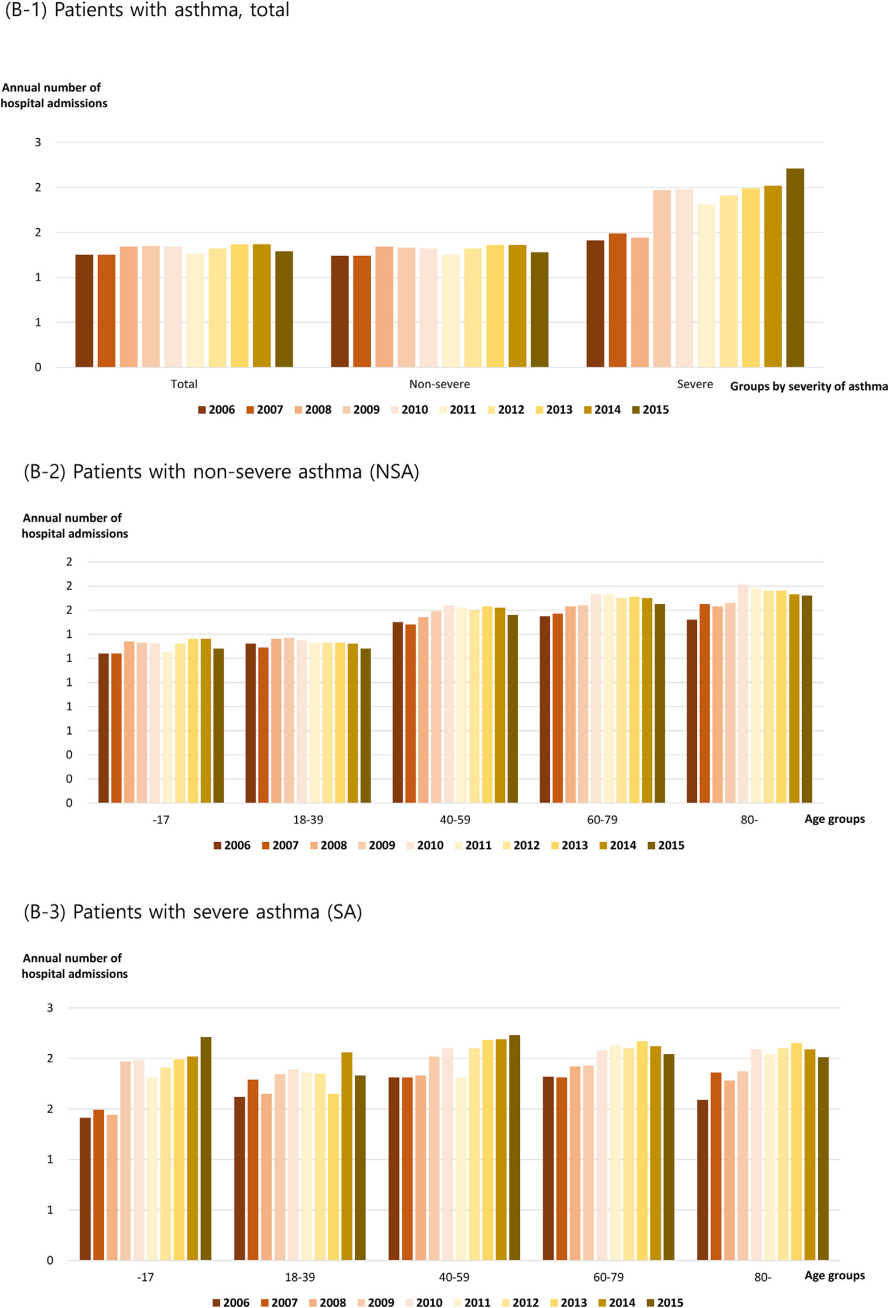
Annual healthcare utilization of patients with asthma. (A) Outpatient setting: (A-1) Patients with asthma, total; (A-2) patients with non-severe asthma (NSA); (A-3) patients with severe asthma (SA). (B) Inpatient setting: (B-1) Patients with asthma, total; (B-2) patients with asthma, non-severe asthma (NSA); (B-3) patients with asthma, severe asthma (SA)

Patients with asthma were categorized according to disease severity. The number of annual asthma-related outpatient visits increased in both groups (NSA and SA) (Fig. 2A). The number of outpatient visits for patients with NSA increased from 4.30 per year in 2006 to 5.03 per year in 2015, while that of patients with SA increased from 10.40 per year in 2006 to 14.23 per year in 2015 (Fig. 2A, Table E3A). The number of annual outpatient visits by patients with NSA was consistently higher among children younger than 18 years and adults older than 60 years than that among other age groups (Fig. 2A, Table E3A). The older age groups had more frequent outpatient visits among adults, and the direct medical cost required per visit was higher, leading to higher overall annual medical costs for older patients (Table E3A). Patients with SA had more annual outpatient visits than patients with NSA in all age groups, and the direct medical cost per visit was also higher. Thus, the annual direct medical cost of patients with SA was higher than that of patients with NSA in all age groups (Fig. 2A, Table E3A).

Between 2005 and 2015, the annual direct cost of outpatient clinic visits due to asthma per patient with NSA and the cost per visit decreased across all age groups (Table E3A). Similarly, the annual direct cost of asthma per patient decreased across all age groups in patients with SA, despite the increase in annual outpatient visits. Moreover, a decrease in cost per visit was observed over 10 years (Table E3A).

The frequency of hospitalization was consistently higher in patients with SA over 10 years (Fig. 2B, Table E3B). The number of annual asthma-related hospitalization days per person in patients with NSA was 8.12 days for children younger than 18 years in 2006 and 8.63 days in 2015. This was the shortest among all age groups for the 10-year period (Table E3B). Patients with NSA aged 80 years or older had more hospitalization days than patients with NSA in the other age groups (23.03 days in 2006 and 34.84 days in 2015). The average number of asthma-related hospitalizations per person per year was 1.52 in 2006 and increased to 1.59 in 2015. Furthermore, there were 1.48 and 1.54 hospitalizations in 2006 and 2015 for patients with NSA, respectively, and 1.79 and 2.07 hospitalizations in 2006 and 2015 for patients with SA, respectively (Table E3B).

### Treatment and prescription patterns according to the severity of asthma

**Fig. 3:**
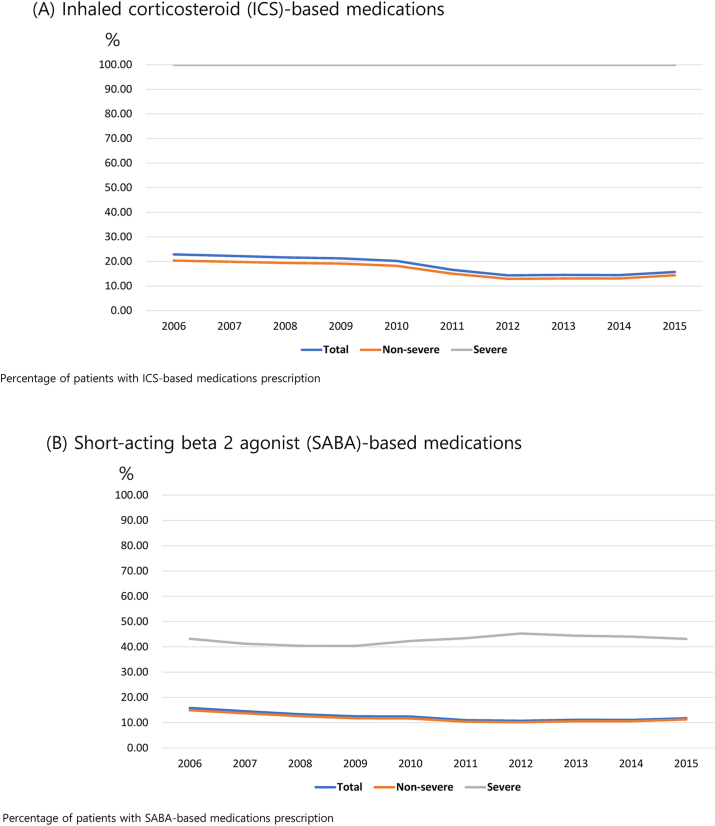

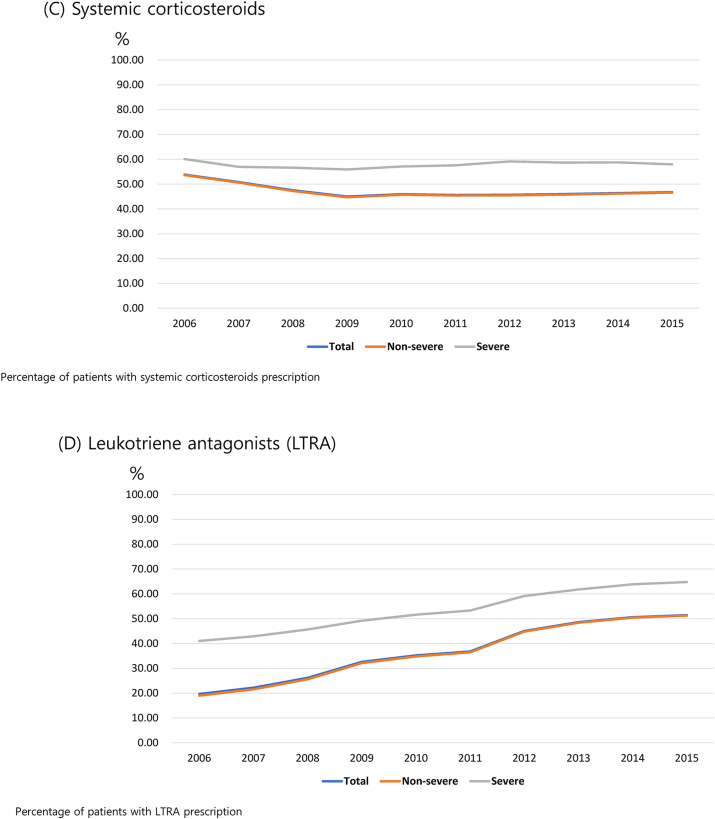
Patterns of asthma-related medication prescription for a 10-year period. (A) Inhaled corticosteroid (ICS)-based medications. (B) Short-acting β-2 agonist (SABA)-based medications. (C) Systemic corticosteroids. (D) Leukotriene antagonists (LTRAs)

The prescription rate of ICSs among patients with asthma decreased from 22.9% in 2006 to 15.7% in 2015. We used the ICS prescription rate to define SA, wherein patients with SA had a rate of 100% (Fig. 3A). The prescription rate of ICSs for children younger than 18 years was consistently the lowest throughout the 10-year period, and the proportion of ICS prescriptions for children in 2015 was only 3.5%. The prescription rate of SABAs was higher in patients with SA than in those with NSA across all age groups, with a prescription rate of >50% in children younger than 18 years with SA during the 10-year period (Table E4). In addition, patients with SA had a higher proportion of those with prescribed systemic corticosteroids across all age groups over the 10-year period compared with patients with NSA (Figure E2C). The prescription rate of LTRAs increased across all patient age groups and was the highest in children younger than 18 years; furthermore, patients with SA had a higher prescription rate of LTRAs than that of patients with NSA over the 10-year period (Figure E2D).

Annual asthma-related test prescriptions, including pulmonary function test (PFT), bronchial provocation test, bronchodilator response test, skin prick test, chest radiography, chest computed tomography (CT), para-nasal sinus (PNS) radiography, multiple allergen simultaneous test (MAST), and serum-specific IgE test (ImmunoCAP), were higher in patients with SA compared with those in patients with NSA (Fig. 4, Table E5).

**Figure:**
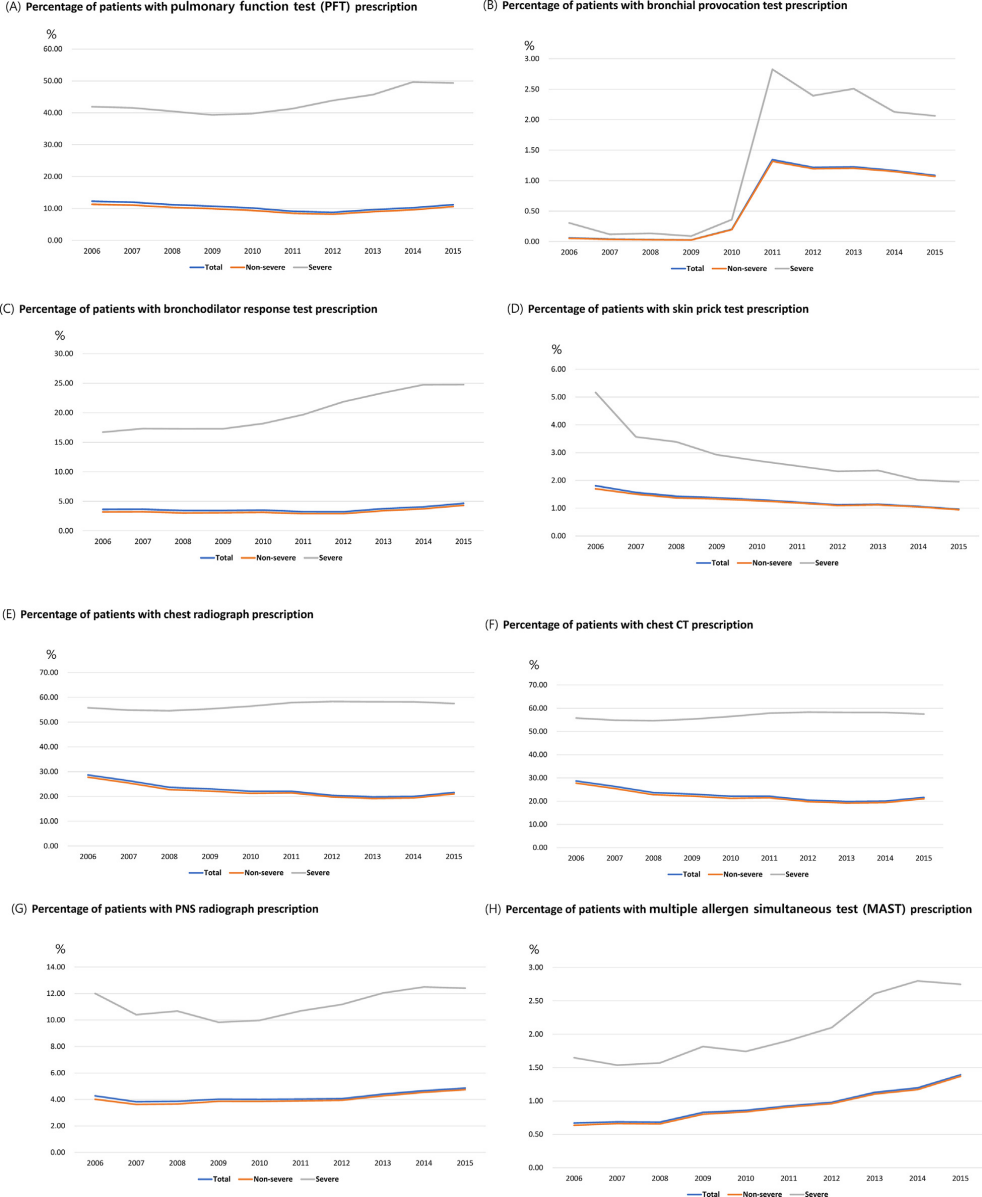

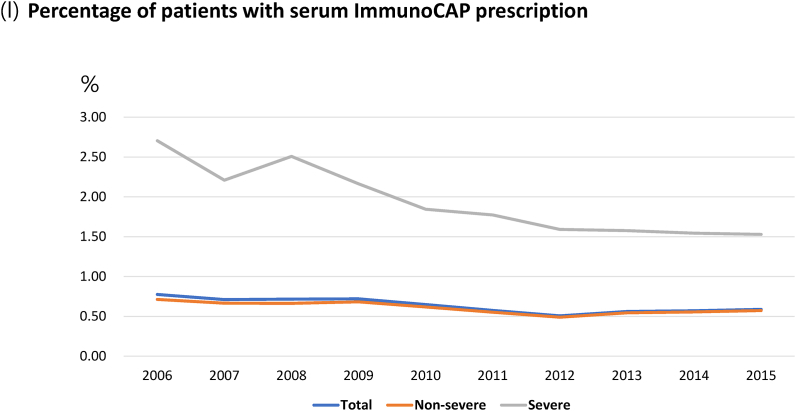

## Discussion

Along with diabetes mellitus and hypertension, asthma is a highly prevalent chronic disease with a significant economic burden. In this study, we analyzed 10 years of NHISS claims data (2006–2015) and classified asthma-related healthcare utilization patterns by disease severity and patient age. Although we did not analyze indirect costs, the economic burden of asthma was the third highest in Korea after diabetes and stroke.^11^, ^12^, ^13^ Inflammatory heart disease and stroke have higher inpatient costs than asthma;^14^ however, our research indicated that the costs of outpatient visits and pharmacotherapy were the major cost burdens associated with asthma. These results are consistent with those of previous studies.^15^, ^16^, ^17^, ^18^ When considered individually, patients who had been hospitalized had inpatient costs as the leading source of cost burden in the annual cost of asthma-related medical expenses, as observed in previous cost-effectiveness studies on asthma.^19^^,^^20^ Therefore, the socioeconomic characteristics of patients who may be expected to have frequent hospitalizations should be considered in clinical practice and management of insurance policies.

SA imposes a significant burden on patients and the socioeconomic system.^21^, ^22^, ^23^ Consistent with our results, in previous studies, patients with difficult-to-treat asthma or SA frequently had a greater economic burden and more recurrent use of healthcare services regardless of the study population.^19^^,^^20^^,^^24^, ^25^, ^26^, ^27^ Although the estimated proportion of patients with SA is only 3−10% of adult patients with asthma worldwide^23^^,^^28^^,^^29^ and 2.5% among all children with asthma,^30^ these patients have increased healthcare costs, reduced quality of life, and increased risk of mortality.^30^, ^31^, ^32^, ^33^, ^34^ However, among patients who had received asthma-related inpatient care, the cost per hospitalization differed slightly between patients with SA and those with NSA. Furthermore, the cost per admission was higher in patients with NSA than in patients with SA between 2011 and 2014. Patients with NSA also had a greater number of annual hospitalization days (data not shown). Therefore, regardless of the severity of asthma, it can be assumed that treatment courses in patients with NSA could be similar to or worse than those in patients with SA once hospitalized.

The prevalence of asthma in 2012 more than tripled than that from 2006, with a rapid annual increase in 2011. This reflects the South Korean government's strengthening of the review and assessment of asthma-related claims, which confirmed the presence or absence of diagnostic codes for asthma in that year. As a result, although the total number of patients with SA increased, the proportion of patients with SA among all patients with asthma decreased. Our study suggests that any socioeconomic factors that could affect claims data during study periods, including changes to national policies or insurance systems, should be considered globally.

In our study, the prescription rate of systemic steroids remained high. Physicians need to carefully consider whether to prescribe systemic corticosteroids to patients with asthma. When controlling asthma symptoms, physicians should adjust ICS doses to levels that will control the patient's symptoms, rather than prescribing systemic corticosteroids, given the potential risks and harms associated with their use.^35^^,^^36^ Although the importance of ICSs in patients with asthma has always been known,^37^, ^38^, ^39^ our study showed a low ICS prescription rate and frequent SABA prescriptions in these patients. To improve the low ICS prescription rate, physicians should focus on controlling asthma using an ICS-based inhaler. Digital healthcare-based patient-customized services, such as portable wireless devices and online applications, which have recently been proposed, can be helpful.^40^, ^41^, ^42^, ^43^ In addition, since our study reflected the clinical practice before the recent revision of the GINA guidelines,^35^ changes are expected in the prescription pattern if claims data are analyzed after revision according to the guidelines.

We believe that our results are interesting because they are from a large-scale, population-based database study covering approximately 98% of the population of the country. To the best of our knowledge, this is the first study utilizing the data of patients with asthma from the entire national population, unlike other previous studies that sampled data.^4^, ^5^, ^6^^,^^44^^,^^45^ Thus, this study provides insights into the status of asthma in the entire population of a country over a long period of 10 years.

However, this study has several limitations. First, it was a retrospective study based on claims data; hence, there is a possibility that errors in diagnostic codes or omission of claims may have occurred. Although these errors may not have been identified, they are a limitation of most claims data analyses. Furthermore, the diagnoses were defined according to a working definition with diagnostic codes and prescription histories rather than by the results of clinical diagnostic tests due to the characteristics of the study. However, we included the prescription of asthma-related drugs in the working definition to increase the accuracy of diagnoses. In addition, because all South Korean citizens are required to enroll in the government-operated healthcare insurance system, the possibility of data omission is less likely than in other countries. This increases the reliability of our research data.

Second, as health insurance and payment of medical expenses are under the control of the government, the decision regarding diagnostic codes or physician-prescribed drugs is usually affected by the national policy at a particular time. Even if the prescription is based on clinical evidence, if the diagnosis or treatment code is not acceptable to the government, the healthcare provider cannot be reimbursed for the medical costs, thereby incurring financial losses. Therefore, physicians could use the diagnostic code for asthma in their prescription to administer treatments that are covered by insurance, even if the symptoms are not clinically appropriate. In these cases, ICSs may not have been prescribed, and the ICS prescription rate may therefore have been lower in all patients with asthma in our study. Similarly, the NHISS database does not include any information on the prescription of biologics because none had been approved as asthma treatments that would be reimbursed by the government until 2015.

Third, we categorized the patients into SA and NSA groups according to the claims data and operational definitions without using clinical parameters such as asthma symptom scores, lung function, or the frequency of systemic steroid use. However, to minimize error, we included the proportion of high-dose ICS prescriptions in the operational definition of SA to represent actual clinical practice. Our study is the first to define SA based on the proportion of high-dose ICSs among studies using large-scale claims data, which reduces the gap between actual clinical data and claims data in our study.

Finally, we analyzed direct costs that reflected medical resources only and did not perform an analysis of indirect costs such as productivity; therefore, an accurate cost analysis was not possible. Previous studies in Korea using similar data reported that the rate of direct costs in the asthma-related socioeconomic burden was approximately five times greater than the rate of indirect costs.^46^ Furthermore, previous studies have revealed that medical and therapeutic medical resource consumption accounted for a major portion of the direct costs of asthma.^13^^,^^46^ Therefore, we calculated the direct costs of medical and therapeutic resources, which can be considered general costs for patients with asthma. In addition, as suggested in previous studies, asthma-related economic costs cannot be compared with those of other countries due to variations in healthcare systems, population characteristics, and provider behavior.^24^ For instance, in our study, the annual direct cost of outpatient visits due to asthma in children in 2006 was 385 USD, which was significantly different from the costs reported in another population-based study (723 USD in 2002).^47^ Indeed, the annual hospitalization costs for admission or emergency room visits for asthma per patient varied from 135 USD^48^ to 733 USD,^49^ and were even reported to account for >3000 USD per person per admission.^19^ Furthermore, other studies conducted in France, Italy, and Spain found that drug costs were lower in Italy than in other countries, while the cost of emergency care was highest in Spain.^24^ Hence, cost-related comparisons among different countries are very limited.

In conclusion, we analyzed 10 years of claims data and identified an increase in the prevalence of asthma and a decrease in the proportion of SA. Patients with SA incurred greater medical costs per person than those with NSA. The pharmaceutical prescription pattern still showed a lack of ICS-based prescriptions, whereas SABAs were prescribed frequently. Since asthma-associated hospitalization is a significant economic burden to an individual, clinicians should pay close attention to the possibility of hospitalization due to exacerbation or loss of asthma control. Although they only represent a small percentage of all patients with asthma, social attention is needed for patients with SA. By comparing the results of this study with the data of future research, after a wide clinical application of the recently changed GINA guidelines,^35^ we will be able to confirm how changes in these clinical guidelines have affected asthma epidemiology and healthcare utilization. However, given the characteristics of claims data, these results should be interpreted considering the changes in the insurance system.

## Abbreviations

NSA, non-severe asthma; SA, severe asthma; NHIS, National Health Insurance System; NHISS, National Health Insurance Sharing Service; ICSs, inhaled corticosteroids; LABAs, long-acting β-2 agonists; LTRAs, leukotriene antagonists; SABAs, short-acting β-2 agonists; PFT, pulmonary function test; MAST, multiple allergen simultaneous test; LAMAs, long-acting muscarinic antagonists; SAMAs, short-acting muscarinic antagonists; CT, computed tomography; PNS, para-nasal sinus.

## Acknowledgement

This study is a collaborative study between the Korean Academy of Asthma, Allergy and Clinical Immunology and the Korean National Health Insurance Service using data from the National Health Insurance Sharing Service (NHISS). The relevant data are available from the Healthcare Bigdata by the NHISS.

## Funding source

This research was supported by a grant of the Korea Health Technology R&D Project through the 10.13039/501100003710Korea Health Industry Development Institute (10.13039/501100003710KHIDI), funded by the 10.13039/100009647Ministry of Health & Welfare, Republic of Korea (grant numbers: HI19C0481, HC20C0076), and a grant from the Korea Asthma Allergy Foundation (KAF).

## Availability of data and materials

The authors confirm that the data supporting the findings of this study are available within the article and its supplementary materials.

## Author contributions

Study design: JH Kim, WJ Song, HS Kwon, YS Cho, YJ Cho, SI Chang, and TB Kim; data collection: JH Kim, HS Lee, SY Park, HJ Kim, JY Kim, SH Choi, and HS Kwon; data analysis: JH Kim, HS Lee, JH Yu, SH Kim, DJ Song, and DH Lim; data interpretation: all authors; writing of the manuscript: JH Kim and TB Kim; reviewing of the manuscript: all authors; final approval of the manuscript: all authors.

## Ethics approval

This study was approved by the Institutional Review Board of the Asan Medical Center (No. S2016-1254-0010) and the Ethics Committee of the National Health Insurance Sharing Service (No. NHIS-2016-4-016).

## Consent for publication

We hereby declare that we all participated in the study and in the development of the manuscript titled “Epidemiology of patients with asthma in Korea: Analysis of the NHISS database from 2006 to 2015”. We have read the final version and provided our consent for the article to be published in the *World Allergy Organization Journal.*

## Potential competing interests

The authors report no competing interests.

## Submission declaration

This manuscript has not been published or presented elsewhere in part or in entirety and is not under consideration by another journal.
