# Supplementary material for: Epidemiology of patients with asthma in Korea: Analysis of the NHISS database 2006–2015
Source: World Allergy Organ J. 2023 Apr 19;16(4):100768. doi: 10.1016/j.waojou.2023.100768 (PMC10148228; doi:10.1016/j.waojou.2023.100768)
Supplement: Multimedia component 1 [file mmc1.doc]

**Table E1. Demographic data of patients with asthma and prevalence of asthma by sex**

|  | **2006** | **2007** | **2008** | **2009** | **2010** | **2011** | **2012** | **2013** | **2014** | **2015** |
| --- | --- | --- | --- | --- | --- | --- | --- | --- | --- | --- |
| Total (n) | 743,968 | 879,501 | 1,043,914 | 1,218,594 | 1,410,370 | 1,955,322 | 2,224,321 | 2,206,624 | 2,347,631 | 2,286,309 |
| Age (mean, years) | 46.5 | 46.3 | 46.5 | 45.5 | 45.2 | 44.2 | 44.0 | 44.4 | 43.9 | 44.6 |
| Age groups (n, %) |  |  |  |  |  |  |  |  |  |  |
| ≤17 | 128,162 (17.2) | 158,696 (18.0) | 196,046 (18.8) | 262,532 (21.5) | 315,624 (22.4) | 454,194 (23.2) | 528,961 (23.8) | 507,114 (23.0) | 570,101 (24.3) | 546,969 (23.9) |
| 18–39 | 126,885 (17.1) | 150,139 (17.1) | 168,715 (16.2) | 193,719 (15.9) | 225,698 (16.0) | 337,030 (17.2) | 370,412 (16.7) | 371,012 (16.8) | 385,194 (16.4) | 353,405 (15.5) |
| 40–59 | 223,176 (30.0) | 258,832 (29.4) | 301,956 (28.9) | 331,588 (27.2) | 376,593 (26.7) | 527,268 (27.0) | 601,819 (27.1) | 604,861 (27.4) | 640,448 (27.3) | 618,567 (27.1) |
| 60–79 | 247,624 (33.3) | 287,110 (32.6) | 342,227 (32.8) | 384,370 (31.5) | 431,756 (30.6) | 548,654 (28.1) | 620,494 (27.9) | 618,706 (28.0) | 638,816 (27.2) | 644,644 (28.2) |
| ≥80 | 18,121 (2.4) | 24,724 (2.8) | 34,970 (3.3) | 46,385 (3.8) | 60,699 (4.3) | 88,176 (4.5) | 102,635 (4.6) | 104,931 (4.8) | 113,072 (4.8) | 122,724 (5.4) |
| Prevalence by age group (%) |  |  |  |  |  |  |  |  |  |  |
| ≤17 | 1.58 | 1.97 | 2.47 | 3.38 | 4.19 | 6.25 | 7.56 | 7.51 | 8.71 | 8.65 |
| 18–39 | 0.72 | 0.86 | 0.99 | 1.15 | 1.36 | 2.05 | 2.29 | 2.32 | 2.43 | 2.25 |
| 40–59 | 1.61 | 1.82 | 2.07 | 2.21 | 2.44 | 3.32 | 3.70 | 3.66 | 3.81 | 3.65 |
| 60–79 | 4.33 | 4.84 | 5.54 | 6.00 | 6.51 | 8.04 | 8.78 | 8.43 | 8.39 | 8.10 |
| ≥80 | 2.62 | 3.37 | 4.46 | 5.49 | 6.69 | 9.10 | 9.90 | 9.40 | 9.37 | 9.35 |
| Total | 1.62 | 1.90 | 2.24 | 2.60 | 2.99 | 4.13 | 4.68 | 4.62 | 4.89 | 4.74 |
| Patients with asthma, female  Age groups (n, %) | | | | | | | | | | |
| ≤17 | 54,513 (12.2) | 66,728 (12.8) | 82,287 (13.3) | 110,970 (15.5) | 134,628 (16.4) | 198,009 (17.5) | 232,572 (18.1) | 222,535 (17.5) | 253,082 (18.7) | 245,092 (18.6) |
| 18–39 | 86,290 (19.4) | 101,941 (19.5) | 113,780 (18.4) | 131,582 (18.4) | 152,877 (18.7) | 224,836 (19.8) | 243,480 (19.0) | 242,498 (19.1) | 250,607 (18.6) | 229,634 (17.4) |
| 40–59 | 142,071 (31.9) | 164,979 (31.5) | 193,496 (31.3) | 213,615 (29.9) | 241,483 (29.5) | 335,306 (29.6) | 380,192 (29.7) | 381,613 (30.1) | 404,925 (30.0) | 390,728 (29.7) |
| 60–79 | 150,103 (33.7) | 172,494 (33.0) | 205,086 (33.2) | 228,136 (31.9) | 250,206 (30.6) | 317,222 (28.0) | 358,311 (28.0) | 355,003 (28.0) | 368,791 (27.3) | 372,636 (28.3) |
| ≥80 | 12,828 (2.9) | 17,191 (3.3) | 23,998 (3.9) | 31,193 (4.4) | 39,804 (4.9) | 57,574 (5.1) | 66,929 (5.2) | 67,773 (5.3) | 73,026 (5.4) | 78,965 (6.0) |
| Total | 445,805 (100.0) | 523,333 (100.0) | 618,647 (100.0) | 715,496 (100.0) | 818,998 (100.0) | 1,132,947 (100.0) | 1,281,484 (100.0) | 1,269,422 (100.0) | 1,350,431 (100.0) | 1,317,055 (100.0) |
| Prevalence by age group (%) | | | | | | | | | | |
| ≤17 | 1.43 | 1.75 | 2.19 | 3.02 | 3.77 | 5.73 | 6.97 | 6.88 | 8.06 | 8.07 |
| 18–39 | 1.01 | 1.20 | 1.37 | 1.61 | 1.90 | 2.83 | 3.11 | 3.14 | 3.28 | 3.04 |
| 40–59 | 2.08 | 2.35 | 2.68 | 2.88 | 3.16 | 4.27 | 4.73 | 4.66 | 4.87 | 4.67 |
| 60–79 | 4.64 | 5.18 | 5.95 | 6.42 | 6.85 | 8.48 | 9.30 | 8.91 | 8.97 | 8.72 |
| ≥80 | 2.62 | 3.30 | 4.30 | 5.19 | 6.16 | 8.34 | 9.09 | 8.60 | 8.62 | 8.64 |
| Total | 1.94 | 2.26 | 2.66 | 3.05 | 3.47 | 4.78 | 5.38 | 5.31 | 5.62 | 5.45 |
| Patients with asthma, male  Age groups (n, %) | | | | | | | | | | |
| ≤17 | 73,649 (24.7) | 91,968 (25.8) | 113,759 (26.8) | 151,562 (30.1) | 180,996 (30.6) | 256,185 (31.2) | 296,389 (31.4) | 284,579 (30.4) | 317,019 (31.8) | 301,877 (31.1) |
| 18–39 | 40,595 (13.6) | 48,198 (13.5) | 54,935 (12.9) | 62,137 (12.4) | 72,821 (12.3) | 112,194 (13.6) | 126,932 (13.5) | 128,514 (13.7) | 134,587 (13.5) | 123,771 (12.8) |
| 40–59 | 81,105 (27.2) | 93,853 (26.4) | 108,460 (25.5) | 117,973 (23.4) | 135,110 (22.8) | 191,962 (23.3) | 221,627 (23.5) | 223,248 (23.8) | 235,523 (23.6) | 227,839 (23.5) |
| 60–79 | 97,521 (32.7) | 114,616 (32.2) | 137,141 (32.2) | 156,234 (31.1) | 181,550 (30.7) | 231,432 (28.1) | 262,183 (27.8) | 263,703 (28.1) | 270,025 (27.1) | 272,008 (28.1) |
| ≥80 | 5,293 (1.8) | 7,533 (2.1) | 10,972 (2.6) | 15,192 (3.0) | 20,895 (3.5) | 30,602 (3.7) | 35,706 (3.8) | 37,158 (4.0) | 40,046 (4.0) | 43,759 (4.5) |
| Total | 298,163 (100.0) | 356,168 (100.0) | 425,267 (100.0) | 503,098 (100.0) | 591,372 (100.0) | 822,375 (100.0) | 942,837 (100.0) | 937,202 (100.0) | 997,200 (100.0) | 969,254 (100.0) |
| Prevalence by age group (%) | | | | | | | | | | |
| ≤17 | 1.72 | 2.16 | 2.72 | 3.71 | 4.57 | 6.72 | 8.10 | 8.08 | 9.31 | 9.19 |
| 18–39 | 0.45 | 0.54 | 0.63 | 0.72 | 0.85 | 1.33 | 1.52 | 1.55 | 1.64 | 1.52 |
| 40–59 | 1.16 | 1.31 | 1.47 | 1.55 | 1.73 | 2.39 | 2.70 | 2.67 | 2.77 | 2.66 |
| 60–79 | 3.92 | 4.41 | 5.02 | 5.47 | 6.10 | 7.51 | 8.17 | 7.86 | 7.72 | 7.39 |
| ≥80 | 2.62 | 3.54 | 4.84 | 6.23 | 7.99 | 10.98 | 11.88 | 11.35 | 11.13 | 10.98 |
| Total | 1.30 | 1.54 | 1.82 | 2.15 | 2.51 | 3.48 | 3.97 | 3.93 | 4.16 | 4.03 |

**Table E2. Healthcare institution visits of patients with asthma by medical specialty**

|  | **2006** | | **2007** | | **2008** | | **2009** | | **2010** | | **2011** | | **2012** | | **2013** | | **2014** | | **2015** | |  |
| --- | --- | --- | --- | --- | --- | --- | --- | --- | --- | --- | --- | --- | --- | --- | --- | --- | --- | --- | --- | --- | --- |
| Total visits | 3,388,201 | (100.0) | 5,058,484 | (100.0) | 6,841,672 | (100.0) | 7,889,745 | (100.0) | 8,777,839 | (100.0) | 10,640,951 | (100.0) | 12,255,754 | (100.0) | 11,996,404 | (100.0) | 12,511,702 | (100.0) | 11,941,270 | (100.0) |  |
| Medical specialty |  |  |  |  |  |  |  |  |  |  |  |  |  |  |  |  |  |  |  |  |  |
| IM |  |  |  |  |  |  |  |  |  |  |  |  |  |  |  |  |  |  |  |  |  |
| Total | 2,363,569 | (69.8) | 3,420,620 | (67.6) | 4,543,654 | (66.4) | 5,099,193 | (64.6) | 5,608,877 | (63.9) | 6,582,966 | (61.9) | 7,193,871 | (58.7) | 6,927,555 | (57.7) | 6,972,718 | (55.7) | 7,305,528 | (61.2) |  |
| ≤17 | 85,218 | (3.6) | 126,948 | (3.7) | 173,141 | (3.8) | 252,225 | (4.9) | 292,041 | (5.2) | 367,003 | (5.6) | 392,759 | (5.5) | 373,008 | (5.4) | 415,928 | (6.0) | 1,004,245 | (13.7) |  |
| 18–39 | 325,004 | (13.8) | 457,439 | (13.4) | 551,964 | (12.1) | 619,229 | (12.1) | 683,116 | (12.2) | 882,641 | (13.4) | 915,790 | (12.7) | 875,664 | (12.6) | 867,116 | (12.4) | 759,631 | (10.4) |  |
| 40–59 | 774,913 | (32.8) | 1,078,409 | (31.5) | 1,374,853 | (30.3) | 1,473,437 | (28.9) | 1,586,905 | (28.3) | 1,886,618 | (28.7) | 2,073,904 | (28.8) | 1,990,551 | (28.7) | 2,007,327 | (28.8) | 1,883,902 | (25.8) |  |
| 60–79 | 1,097,260 | (46.4) | 1,614,063 | (47.2) | 2,204,449 | (48.5) | 2,436,757 | (47.8) | 2,643,410 | (47.1) | 2,941,802 | (44.7) | 3,235,823 | (45.0) | 3,106,184 | (44.8) | 3,072,213 | (44.1) | 3,010,612 | (41.2) |  |
| ≥80 | 81,174 | (3.4) | 143,761 | (4.2) | 239,247 | (5.3) | 317,545 | (6.2) | 403,405 | (7.2) | 504,902 | (7.7) | 575,595 | (8.0) | 582,148 | (8.4) | 610,134 | (8.8) | 647,138 | (8.9) |  |
| ENT |  |  |  |  |  |  |  |  |  |  |  |  |  |  |  |  |  |  |  |  |  |
| Total | 214,634 | (6.3) | 361,610 | (7.1) | 564,696 | (8.3) | 687,189 | (8.7) | 804,583 | (9.2) | 1,125,799 | (10.6) | 1,596,123 | (13.0) | 1,818,574 | (15.2) | 2,118,921 | (16.9) | 2,069,260 | (17.3) |  |
| ≤17 | 55,065 | (25.7) | 93,825 | (25.9) | 148,821 | (26.4) | 204,634 | (29.8) | 250,066 | (31.1) | 331,557 | (29.5) | 483,114 | (30.3) | 549,872 | (30.2) | 669,490 | (31.6) | 629,589 | (30.4) |  |
| 18–39 | 52,299 | (24.4) | 84,871 | (23.5) | 122,958 | (21.8) | 139,922 | (20.4) | 164,090 | (20.4) | 240,711 | (21.4) | 324,329 | (20.3) | 370,576 | (20.4) | 418,026 | (19.7) | 387,753 | (18.7) |  |
| 40–59 | 63,021 | (29.4) | 104,853 | (29.0) | 162,120 | (28.7) | 182,887 | (26.6) | 209,837 | (26.1) | 301,397 | (26.8) | 424,187 | (26.6) | 482,664 | (26.5) | 557,600 | (26.3) | 558,193 | (27.0) |  |
| 60–79 | 42,596 | (19.8) | 75,025 | (20.7) | 124,360 | (22.0) | 150,659 | (21.9) | 168,853 | (21.0) | 233,108 | (20.7) | 334,455 | (21.0) | 378,166 | (20.8) | 428,598 | (20.2) | 441,531 | (21.3) |  |
| ≥80 | 1,653 | (0.8) | 3,036 | (0.8) | 6,437 | (1.1) | 9,087 | (1.3) | 11,737 | (1.5) | 19,026 | (1.7) | 30,038 | (1.9) | 37,296 | (2.1) | 45,207 | (2.1) | 52,194 | (2.5) |  |
| PED |  |  |  |  |  |  |  |  |  |  |  |  |  |  |  |  |  |  |  |  |  |
| Total | 363,637 | (10.7) | 580,506 | (11.5) | 797,274 | (11.7) | 1,039,273 | (13.2) | 1,183,666 | (13.5) | 1,513,968 | (14.2) | 1,895,643 | (15.5) | 1,774,443 | (14.8) | 1,933,891 | (15.5) | 1,180,268 | (9.9) |  |
| ≤17 | 338,916 | (93.2) | 542,743 | (93.5) | 745,976 | (93.6) | 983,416 | (94.6) | 1,121,787 | (94.8) | 1,428,785 | (94.4) | 1,799,094 | (94.9) | 1,681,736 | (94.8) | 1,835,329 | (94.9) | 1,061,230 | (89.9) |  |
| 18–39 | 9,537 | (2.6) | 14,112 | (2.4) | 17,272 | (2.2) | 19,184 | (1.8) | 22,623 | (1.9) | 31,147 | (2.1) | 36,488 | (1.9) | 36,007 | (2.0) | 37,256 | (1.9) | 45,182 | (3.8) |  |
| 40–59 | 7,640 | (2.1) | 11,379 | (2.0) | 16,171 | (2.0) | 17,450 | (1.7) | 18,231 | (1.5) | 25,149 | (1.7) | 28,614 | (1.5) | 27,787 | (1.6) | 30,847 | (1.6) | 38,834 | (3.3) |  |
| 60–79 | 7,126 | (2.0) | 11,417 | (2.0) | 16,275 | (2.0) | 17,342 | (1.7) | 18,925 | (1.6) | 25,408 | (1.7) | 27,667 | (1.5) | 25,212 | (1.4) | 25,589 | (1.3) | 29,932 | (2.5) |  |
| ≥80 | 418 | (0.1) | 855 | (0.1) | 1,580 | (0.2) | 1,881 | (0.2) | 2,100 | (0.2) | 3,479 | (0.2) | 3,780 | (0.2) | 3,701 | (0.2) | 4,870 | (0.3) | 5,090 | (0.4) |  |
| FM |  |  |  |  |  |  |  |  |  |  |  |  |  |  |  |  |  |  |  |  |  |
| Total | 117,827 | (3.5) | 188,157 | (3.7) | 255,965 | (3.7) | 294,442 | (3.7) | 338,287 | (3.9) | 412,947 | (3.9) | 464,363 | (3.8) | 434,322 | (3.6) | 452,477 | (3.6) | 420,324 | (3.5) |  |
| ≤17 | 12,696 | (10.8) | 17,508 | (9.3) | 22,494 | (8.8) | 32,938 | (11.2) | 40,281 | (11.9) | 52,038 | (12.6) | 61,691 | (13.3) | 54,577 | (12.6) | 63,345 | (14.0) | 54,560 | (13.0) |  |
| 18–39 | 12,754 | (10.8) | 17,435 | (9.3) | 19,685 | (7.7) | 23,248 | (7.9) | 27,191 | (8.0) | 36,333 | (8.8) | 38,943 | (8.4) | 33,747 | (7.8) | 35,036 | (7.7) | 30,665 | (7.3) |  |
| 40–59 | 33,491 | (28.4) | 48,233 | (25.6) | 64,789 | (25.3) | 68,204 | (23.2) | 77,692 | (23.0) | 95,867 | (23.2) | 107,084 | (23.1) | 100,361 | (23.1) | 105,019 | (23.2) | 98,689 | (23.5) |  |
| 60–79 | 53,782 | (45.6) | 94,126 | (50.0) | 129,173 | (50.5) | 144,073 | (48.9) | 159,657 | (47.2) | 185,883 | (45.0) | 206,943 | (44.6) | 195,577 | (45.0) | 195,697 | (43.3) | 183,544 | (43.7) |  |
| ≥80 | 5,104 | (4.3) | 10,855 | (5.8) | 19,824 | (7.7) | 25,979 | (8.8) | 33,466 | (9.9) | 42,826 | (10.4) | 49,702 | (10.7) | 50,060 | (11.5) | 53,380 | (11.8) | 52,866 | (12.6) |  |
| GP |  |  |  |  |  |  |  |  |  |  |  |  |  |  |  |  |  |  |  |  |  |
| Total | 53,558 | (1.6) | 57,212 | (1.1) | 59,834 | (0.9) | 62,459 | (0.8) | 59,140 | (0.7) | 53,497 | (0.5) | 53,613 | (0.4) | 46,078 | (0.4) | 41,687 | (0.3) | 34,360 | (0.3) |  |
| ≤17 | 211 | (0.4) | 287 | (0.5) | 314 | (0.5) | 666 | (1.1) | 286 | (0.5) | 314 | (0.6) | 473 | (0.9) | 418 | (0.9) | 611 | (1.5) | 428 | (1.2) |  |
| 18–39 | 827 | (1.5) | 940 | (1.6) | 942 | (1.6) | 1,076 | (1.7) | 813 | (1.4) | 875 | (1.6) | 1,110 | (2.1) | 845 | (1.8) | 727 | (1.7) | 683 | (2.0) |  |
| 40–59 | 7,517 | (14.0) | 8,016 | (14.0) | 8,178 | (13.7) | 7,369 | (11.8) | 6,324 | (10.7) | 5,701 | (10.7) | 6,112 | (11.4) | 5,275 | (11.4) | 4,930 | (11.8) | 3,639 | (10.6) |  |
| 60–79 | 39,879 | (74.5) | 41,740 | (73.0) | 42,527 | (71.1) | 43,808 | (70.1) | 40,399 | (68.3) | 35,950 | (67.2) | 35,079 | (65.4) | 30,355 | (65.9) | 26,941 | (64.6) | 21,966 | (63.9) |  |
| ≥80 | 5,124 | (9.6) | 6,229 | (10.9) | 7,873 | (13.2) | 9,540 | (15.3) | 11,318 | (19.1) | 10,657 | (19.9) | 10,839 | (20.2) | 9,185 | (19.9) | 8,478 | (20.3) | 7,644 | (22.2) |  |
| ETC |  |  |  |  |  |  |  |  |  |  |  |  |  |  |  |  |  |  |  |  |  |
| Total | 274,976 | (8.1) | 450,379 | (8.9) | 620,249 | (9.1) | 707,189 | (9.0) | 783,286 | (8.9) | 951,774 | (8.9) | 1,052,141 | (8.6) | 995,432 | (8.3) | 992,008 | (7.9) | 931,530 | (7.8) |  |
| ≤17 | 12,970 | (4.7) | 19,565 | (4.3) | 25,429 | (4.1) | 34,229 | (4.8) | 42,403 | (5.4) | 71,959 | (7.6) | 94,657 | (9.0) | 91,153 | (9.2) | 87,389 | (8.8) | 73,427 | (7.9) |  |
| 18–39 | 19,031 | (6.9) | 27,866 | (6.2) | 32,853 | (5.3) | 36,182 | (5.1) | 41,732 | (5.3) | 57,895 | (6.1) | 67,366 | (6.4) | 63,150 | (6.3) | 63,253 | (6.4) | 54,149 | (5.8) |  |
| 40–59 | 67,143 | (24.4) | 98,827 | (21.9) | 126,637 | (20.4) | 135,534 | (19.2) | 146,664 | (18.7) | 183,949 | (19.3) | 214,383 | (20.4) | 204,254 | (20.5) | 207,325 | (20.9) | 191,816 | (20.6) |  |
| 60–79 | 157,995 | (57.5) | 269,588 | (59.9) | 376,939 | (60.8) | 422,593 | (59.8) | 452,648 | (57.8) | 509,552 | (53.5) | 537,914 | (51.1) | 502,243 | (50.5) | 492,493 | (49.6) | 468,936 | (50.3) |  |
| ≥80 | 17,837 | (6.5) | 34,533 | (7.7) | 58,391 | (9.4) | 78,651 | (11.1) | 99,839 | (12.7) | 128,419 | (13.5) | 137,821 | (13.1) | 134,632 | (13.5) | 141,548 | (14.3) | 143,202 | (15.4) |  |

*IM, internal medicine; ENT, otorhinolaryngology; PED, pediatrics; FM, family medicine; GP, general physician; ETC, sum of all other specialties.

**Table E3.** Healthcare utilization of patients with asthma by asthma severity. (A) Outpatient setting. (B) Inpatient setting

|  | | **2006** | | **2007** | | **2008** | | **2009** | | **2010** | | **2011** | | **2012** | | **2013** | | **2014** | | **2015** | |
| --- | --- | --- | --- | --- | --- | --- | --- | --- | --- | --- | --- | --- | --- | --- | --- | --- | --- | --- | --- | --- | --- |
| **Annual outpatient visits per person** | | | | | | | | | | | | | | | | | | | | | |
| Non-severe | 4.30 | | 5.49 | | 6.27 | | 6.20 | | 5.96 | | 5.23 | | 5.31 | | 5.24 | | 5.14 | | 5.03 | |  |
| Severe | 10.40 | | 12.41 | | 14.27 | | 14.32 | | 14.28 | | 14.05 | | 14.86 | | 14.51 | | 14.43 | | 14.23 | |  |
| Cost per year^1)^ | | | | | | | | | | | | | | | | | | | | |  |
| Non-severe | 292 | | 169 | | 142 | | 125 | | 139 | | 120 | | 99 | | 99 | | 99 | | 97 | |  |
| Severe | 1,033 | | 1,093 | | 957 | | 857 | | 996 | | 1,026 | | 924 | | 899 | | 887 | | 822 | |  |
| Cost per visit^2)^ | | | | | | | | | | | | | | | | | | | | | |
| Non-severe | 39 | | 33 | | 24 | | 22 | | 25 | | 25 | | 20 | | 20 | | 21 | | 21 | |  |
| Severe | 104 | | 94 | | 73 | | 66 | | 77 | | 82 | | 70 | | 69 | | 68 | | 64 | |  |
| **Patients with non-severe asthma** | | | | | | | | | | | | | | | | | | | | | |
| **Annual outpatient visits per person by age group** | | | | | | | | | | | | | | | | | | | | | |
| ≤17 | | 3.88 | | 4.99 | | 5.64 | | 5.69 | | 5.48 | | 4.91 | | 5.31 | | 5.39 | | 5.34 | | 5.10 | |
| 18–39 | | 3.16 | | 3.87 | | 4.28 | | 4.21 | | 4.06 | | 3.64 | | 3.67 | | 3.65 | | 3.63 | | 3.55 | |
| 40–59 | | 3.98 | | 4.92 | | 5.50 | | 5.40 | | 5.17 | | 4.53 | | 4.55 | | 4.45 | | 4.37 | | 4.31 | |
| 60–79 | | 5.30 | | 6.97 | | 8.04 | | 7.95 | | 7.65 | | 6.79 | | 6.69 | | 6.49 | | 6.29 | | 6.11 | |
| ≥80 | | 5.80 | | 7.78 | | 9.21 | | 9.19 | | 8.89 | | 7.73 | | 7.55 | | 7.45 | | 7.31 | | 7.08 | |
| **Cost per year^1)^** | | | | | | | | | | | | | | | | | | | | | |
| ≤17 | | 71 | | 69 | | 57 | | 50 | | 55 | | 52 | | 44 | | 42 | | 44 | | 40 | |
| 18–39 | | 825 | | 84 | | 71 | | 61 | | 65 | | 57 | | 47 | | 46 | | 47 | | 47 | |
| 40–59 | | 160 | | 163 | | 133 | | 118 | | 128 | | 108 | | 89 | | 86 | | 87 | | 85 | |
| 60–79 | | 251 | | 267 | | 224 | | 204 | | 231 | | 208 | | 171 | | 170 | | 171 | | 164 | |
| ≥80 | | 269 | | 301 | | 268 | | 254 | | 298 | | 275 | | 227 | | 235 | | 243 | | 233 | |
| **Cost per visit^2)^** | | | | | | | | | | | | | | | | | | | | | |
| ≤17 | | 19 | | 14 | | 10 | | 9 | | 10 | | 11 | | 8 | | 8 | | 8 | | 8 | |
| 18–39 | | 27 | | 22 | | 17 | | 15 | | 17 | | 16 | | 13 | | 13 | | 14 | | 14 | |
| 40–59 | | 41 | | 35 | | 25 | | 23 | | 26 | | 25 | | 21 | | 21 | | 21 | | 21 | |
| 60–79 | | 49 | | 41 | | 31 | | 28 | | 33 | | 34 | | 28 | | 29 | | 30 | | 29 | |
| ≥80 | | 49 | | 42 | | 33 | | 31 | | 38 | | 40 | | 34 | | 35 | | 37 | | 37 | |
| Total | | 39 | | 33 | | 24 | | 22 | | 25 | | 25 | | 20 | | 20 | | 21 | | 21 | |
| **Patients with severe asthma** | | | | | | | | | | | | | | | | | | | | | |
| **Annual outpatient visits per person by age group** | | | | | | | | | | | | | | | | | | | | | |
| ≤17 | | 8.52 | | 10.24 | | 11.63 | | 12.29 | | 12.24 | | 12.22 | | 14.58 | | 13.42 | | 14.32 | | 14.12 | |
| 18–39 | | 9.77 | | 11.46 | | 12.67 | | 12.51 | | 12.55 | | 12.40 | | 13.33 | | 12.75 | | 13.00 | | 13.00 | |
| 40–59 | | 10.30 | | 12.03 | | 13.64 | | 13.60 | | 13.67 | | 13.50 | | 14.36 | | 14.23 | | 14.02 | | 13.87 | |
| 60–79 | | 10.71 | | 12.94 | | 15.03 | | 15.03 | | 14.90 | | 14.60 | | 15.39 | | 14.91 | | 14.91 | | 14.68 | |
| ≥80 | | 10.53 | | 12.38 | | 14.07 | | 14.38 | | 14.01 | | 13.76 | | 14.04 | | 14.18 | | 13.67 | | 13.47 | |
| Cost per year^1)^ | | | | | | | | | | | | | | | | | | | | | |
| ≤17 | | 419 | | 456 | | 363 | | 343 | | 428 | | 404 | | 365 | | 362 | | 363 | | 385 | |
| 18–39 | | 803 | | 803 | | 679 | | 609 | | 693 | | 716 | | 649 | | 633 | | 606 | | 549 | |
| 40–59 | | 1,013 | | 1,052 | | 908 | | 794 | | 923 | | 957 | | 855 | | 825 | | 819 | | 747 | |
| 60–79 | | 1,127 | | 1,194 | | 1,045 | | 934 | | 1,077 | | 1,102 | | 988 | | 961 | | 950 | | 879 | |
| ≥80 | | 1,087 | | 1,224 | | 1,072 | | 956 | | 1,100 | | 1,105 | | 1,003 | | 987 | | 960 | | 901 | |
| Cost per visit^2)^ | | | | | | | | | | | | | | | | | | | | | |
| ≤17 | | 52 | | 47 | | 34 | | 30 | | 38 | | 36 | | 28 | | 29 | | 27 | | 30 | |
| 18–39 | | 86 | | 74 | | 57 | | 51 | | 58 | | 62 | | 53 | | 54 | | 50 | | 46 | |
| 40–59 | | 102 | | 93 | | 72 | | 63 | | 74 | | 78 | | 66 | | 64 | | 64 | | 60 | |
| 60–79 | | 110 | | 100 | | 77 | | 69 | | 81 | | 85 | | 73 | | 73 | | 71 | | 67 | |
| ≥80 | | 107 | | 107 | | 85 | | 74 | | 88 | | 92 | | 82 | | 79 | | 79 | | 75 | |

(B)

|  | **2006** | **2007** | **2008** | **2009** | **2010** | **2011** | **2012** | **2013** | **2014** | **2015** |
| --- | --- | --- | --- | --- | --- | --- | --- | --- | --- | --- |
| **Annual number of hospital admissions** | | | | | | | | | | |
| Non-severe | 1.48 | 1.49 | 1.56 | 1.57 | 1.64 | 1.62 | 1.62 | 1.63 | 1.61 | 1.54 |
| Severe | 1.79 | 1.81 | 1.86 | 1.94 | 2.08 | 2.04 | 2.09 | 2.14 | 2.13 | 2.07 |
| **Cost of hospital admission per person per year^1)^** | | | | | | | | | | |
| Non-severe | 2,154 | 2,528 | 2,377 | 2,227 | 2,788 | 3,525 | 3,326 | 3,478 | 3,483 | 3,223 |
| Severe | 2,795 | 3,413 | 3,230 | 2,934 | 3,617 | 4,197 | 4,017 | 4,300 | 4,582 | 4,383 |
| **Cost per person per admission^2)^** | | | | | | | | | | |
| Non-severe | 1,453 | 1,696 | 1,519 | 1,419 | 1,699 | 2,171 | 2,050 | 2,131 | 2,169 | 2,090 |
| Severe | 1,565 | 1,888 | 1,729 | 1,510 | 1,744 | 2,004 | 1,923 | 2,005 | 2,153 | 2,116 |
| **Hospitalization days** | | | | | | | | | | |
| Per person per year (mean) | | | | | | | | | | |
| Non-severe | 17.09 | 17.05 | 17.94 | 17.83 | 18.56 | 18.75 | 17.64 | 17.87 | 16.61 | 15.06 |
| Severe | 19.40 | 20.96 | 21.90 | 22.04 | 23.41 | 24.76 | 23.43 | 24.31 | 24.39 | 22.35 |
| Per admission (mean) | | | | | | | | | | |
| Non-severe | 12.56 | 12.99 | 13.29 | 13.54 | 14.14 | 15.42 | 15.05 | 15.12 | 14.46 | 13.89 |
| Severe | 11.25 | 12.12 | 12.64 | 12.53 | 12.89 | 13.63 | 13.35 | 13.53 | 13.92 | 13.20 |
| **Patients with non-severe asthma** | | | | | | | | | | |
| **Annual number of hospital admissions by age group** | | | | | | | | | | |
| ≤17 | 1.24 | 1.24 | 1.34 | 1.33 | 1.32 | 1.25 | 1.32 | 1.36 | 1.36 | 1.28 |
| 18–39 | 1.32 | 1.29 | 1.36 | 1.37 | 1.35 | 1.33 | 1.33 | 1.33 | 1.32 | 1.28 |
| 40–59 | 1.50 | 1.48 | 1.54 | 1.59 | 1.64 | 1.62 | 1.60 | 1.63 | 1.62 | 1.56 |
| 60–79 | 1.55 | 1.57 | 1.63 | 1.64 | 1.73 | 1.73 | 1.70 | 1.71 | 1.70 | 1.65 |
| ≥80 | 1.52 | 1.65 | 1.63 | 1.66 | 1.81 | 1.78 | 1.76 | 1.76 | 1.73 | 1.72 |
| Cost per year^1)^ | | | | | | | | | | |
| ≤17 | 953 | 1,090 | 1,065 | 1,014 | 1,108 | 1,087 | 1,171 | 1,196 | 1,140 | 1,083 |
| 18–39 | 1,484 | 1,636 | 1,530 | 1,363 | 1,626 | 1,985 | 1,671 | 1,757 | 2,059 | 1,919 |
| 40–59 | 2,170 | 2,420 | 2,266 | 2,095 | 2,525 | 3,149 | 2,898 | 3,013 | 3,037 | 2,947 |
| 60–79 | 2,494 | 2,997 | 2,736 | 2,664 | 3,322 | 4,311 | 3,918 | 4,129 | 4,272 | 4,127 |
| ≥80 | 2,458 | 3,166 | 2,733 | 2,700 | 3,642 | 4,551 | 4,326 | 4,655 | 4,989 | 4,702 |
| Cost per admission^2)^ | | | | | | | | | | |
| ≤17 | 767 | 876 | 795 | 761 | 838 | 870 | 889 | 877 | 837 | 846 |
| 18–39 | 1,129 | 1,266 | 1,121 | 991 | 1,200 | 1,492 | 1,258 | 1,317 | 1,563 | 1,503 |
| 40–59 | 1,448 | 1,638 | 1,467 | 1,314 | 1,540 | 1,948 | 1,811 | 1,850 | 1,875 | 1,893 |
| 60–79 | 1,606 | 1,912 | 1,675 | 1,628 | 1,922 | 2,486 | 2,301 | 2,419 | 2,519 | 2,498 |
| ≥80 | 1,614 | 1,913 | 1,672 | 1,623 | 2,011 | 2,560 | 2,465 | 2,651 | 2,882 | 2,741 |
| **Patients with severe asthma** | | | | | | | | | | |
| **Annual number of hospital admissions by age group** | | | | | | | | | | |
| ≤17 | 1.41 | 1.49 | 1.44 | 1.97 | 1.98 | 1.81 | 1.91 | 1.99 | 2.02 | 2.21 |
| 18–39 | 1.62 | 1.79 | 1.65 | 1.84 | 1.89 | 1.86 | 1.85 | 1.65 | 2.06 | 1.83 |
| 40–59 | 1.81 | 1.81 | 1.83 | 2.02 | 2.10 | 1.81 | 2.10 | 2.18 | 2.19 | 2.23 |
| 60–79 | 1.82 | 1.81 | 1.92 | 1.93 | 2.08 | 2.13 | 2.10 | 2.17 | 2.12 | 2.04 |
| ≥80 | 1.59 | 1.86 | 1.78 | 1.87 | 2.09 | 2.04 | 2.10 | 2.15 | 2.09 | 2.01 |
| Cost per year^1)^ | | | | | | | | | | |
| ≤17 | 967 | 1,333 | 1,631 | 1,578 | 1,730 | 1,550 | 1,497 | 2,506 | 2,156 | 2,194 |
| 18–39 | 1,957 | 3,181 | 2,135 | 2,152 | 2,439 | 2,825 | 2,426 | 2,499 | 3,585 | 2,454 |
| 40–59 | 2,721 | 3,230 | 2,982 | 2,835 | 3,329 | 3,620 | 3,419 | 3,713 | 3,935 | 4,113 |
| 60–79 | 3,027 | 3,597 | 3,476 | 3,026 | 3,804 | 4,513 | 4,249 | 4,528 | 4,795 | 4,503 |
| ≥80 | 2,804 | 3,335 | 3,291 | 3,299 | 3,946 | 4,373 | 4,618 | 5,005 | 5,289 | 4,884 |
| Cost per admission^2)^ | | | | | | | | | | |
| ≤17 | 685 | 896 | 1,134 | 799 | 875 | 858 | 784 | 1,261 | 1,065 | 994 |
| 18–39 | 1,208 | 1,777 | 1,296 | 1,169 | 1,290 | 1,519 | 1,310 | 1,512 | 1,742 | 1,338 |
| 40–59 | 1,500 | 1,782 | 1,627 | 1,405 | 1,588 | 1,743 | 1,624 | 1,701 | 1,793 | 1,847 |
| 60–79 | 1,665 | 1,986 | 1,806 | 1,567 | 1,831 | 2,115 | 2,024 | 2,086 | 2,264 | 2,203 |
| ≥80 | 1,758 | 1,792 | 1,846 | 1,763 | 1,885 | 2,143 | 2,195 | 2,328 | 2,526 | 2,428 |

1. Direct medical costs per person per year.
2. Direct medical costs per person per hospitalization or outpatient visit.
3. *All costs are presented in USD as the average annual exchange rate for each year (1 USD=955,74 Korean won [KRW] in 2006, 1 USD=929.20 KRW in 2007, 1 USD=1,101.93 KRW in 2008, 1 USD=1,276.18 KRW in 2009, 1 USD=1,156.06 KRW in 2010, 1 USD=1,108.09 KRW in 2011, 1 USD=1,126.43 KRW in 2012, 1 USD=1,094.97 KRW in 2013, 1 USD=1,053.30 KRW in 2014, and 1 USD=1,132.10 KRW in 2015).

**Table E4.** Patterns of asthma-related medication prescription over a 10-year period

|  | **2006** | **2007** | **2008** | **2009** | **2010** | **2011** | **2012** | **2013** | **2014** | **2015** |
| --- | --- | --- | --- | --- | --- | --- | --- | --- | --- | --- |
| Inhaled corticosteroid (ICS)-based medications | | | | | | | | | | |
| Total (%) | 22.86 | 22.26 | 21.64 | 21.27 | 20.20 | 16.57 | 14.34 | 14.51 | 14.41 | 15.70 |
| Non-severe (%) | 20.36 | 19.88 | 19.37 | 19.13 | 18.22 | 15.00 | 12.92 | 13.06 | 13.07 | 14.38 |
| Severe (%) | 100.00 | 100.00 | 100.00 | 100.00 | 100.00 | 100.00 | 100.00 | 100.00 | 100.00 | 100.00 |
| Short-acting beta-2 agonist (SABA)-based medications | | | | | | | | | | |
| Total (%) | 15.81 | 14.52 | 13.34 | 12.52 | 12.44 | 11.00 | 10.75 | 11.16 | 11.10 | 11.78 |
| Non-severe (%) | 14.91 | 13.70 | 12.55 | 11.76 | 11.69 | 10.38 | 10.17 | 10.59 | 10.58 | 11.28 |
| Severe (%) | 43.17 | 41.20 | 40.40 | 40.35 | 42.31 | 43.39 | 45.24 | 44.40 | 44.03 | 43.13 |
| Systemic corticosteroids | | | | | | | | | | |
| Total (%) | 53.79 | 50.79 | 47.49 | 44.98 | 45.95 | 45.61 | 45.67 | 45.96 | 46.38 | 46.73 |
| Non-severe (%) | 53.58 | 50.60 | 47.23 | 44.68 | 45.67 | 45.38 | 45.45 | 45.74 | 46.18 | 46.55 |
| Severe (%) | 60.07 | 56.93 | 56.62 | 55.87 | 57.08 | 57.55 | 59.12 | 58.64 | 58.73 | 57.94 |
| Leukotriene antagonists (LTRAs) | | | | | | | | | | |
| Total (%) | 19.68 | 22.18 | 26.16 | 32.58 | 35.19 | 36.76 | 45.04 | 48.60 | 50.60 | 51.41 |
| Non-severe (%) | 18.98 | 21.55 | 25.60 | 32.13 | 34.78 | 36.44 | 44.80 | 48.37 | 50.39 | 51.19 |
| Severe (%) | 41.00 | 42.87 | 45.65 | 49.15 | 51.59 | 53.27 | 59.14 | 61.77 | 63.87 | 64.77 |

**Table E5. Patterns of asthma-related medical exam prescriptions over a 10-year period**

|  | **2006** | **2007** | **2008** | **2009** | **2010** | **2011** | **2012** | **2013** | **2014** | **2015** |
| --- | --- | --- | --- | --- | --- | --- | --- | --- | --- | --- |
| **Pulmonary function test (PFT)**  **Percentage of patients with a PFT prescription** | | | | | | | | | | |
| Total (%) | 12.28 | 11.96 | 11.18 | 10.71 | 10.13 | 9.11 | 8.80 | 9.62 | 10.22 | 11.19 |
| Non-severe (%) | 11.32 | 11.04 | 10.33 | 9.93 | 9.39 | 8.50 | 8.21 | 9.01 | 9.59 | 10.59 |
| Severe (%) | 41.89 | 41.55 | 40.47 | 39.32 | 39.77 | 41.29 | 43.84 | 45.69 | 49.65 | 49.32 |
| **Number of prescriptions with a PFT per patient per year** | | | | | | | | | | |
| Total | 1.50 | 1.48 | 1.47 | 1.44 | 1.43 | 1.37 | 1.37 | 1.38 | 1.37 | 1.36 |
| Non-severe | 1.37 | 1.37 | 1.37 | 1.36 | 1.36 | 1.31 | 1.31 | 1.32 | 1.32 | 1.31 |
| Severe | 2.62 | 2.44 | 2.34 | 2.19 | 2.09 | 2.03 | 2.02 | 2.06 | 2.01 | 1.98 |
| **Bronchial provocation test**  **Percentage of patients with a bronchial provocation test prescription** | | | | | | | | | | |
| Total (%) | 0.06 | 0.04 | 0.03 | 0.03 | 0.20 | 1.34 | 1.22 | 1.23 | 1.17 | 1.08 |
| Non-severe (%) | 0.05 | 0.04 | 0.03 | 0.03 | 0.20 | 1.32 | 1.20 | 1.20 | 1.15 | 1.07 |
| Severe (%) | 0.31 | 0.12 | 0.14 | 0.09 | 0.36 | 2.83 | 2.39 | 2.51 | 2.13 | 2.06 |
| **Number of prescriptions with a bronchial provocation test per patient per year** | | | | | | | | | | |
| Total | 1.02 | 1.01 | 1.02 | 1.01 | 1.01 | 1.04 | 1.03 | 1.03 | 1.03 | 1.02 |
| Non-severe | 1.02 | 1.01 | 1.03 | 1.01 | 1.01 | 1.04 | 1.03 | 1.03 | 1.02 | 1.02 |
| Severe | 1.06 | 1.00 | 1.03 | 1.00 | 1.02 | 1.09 | 1.07 | 1.08 | 1.09 | 1.04 |
| **Bronchodilator response test**  **Percentage of patients with a bronchodilator response test prescription** | | | | | | | | | | |
| Total (%) | 3.63 | 3.66 | 3.44 | 3.45 | 3.51 | 3.25 | 3.24 | 3.75 | 4.07 | 4.65 |
| Non-severe (%) | 3.21 | 3.24 | 3.04 | 3.07 | 3.14 | 2.93 | 2.93 | 3.41 | 3.74 | 4.33 |
| Severe (%) | 16.71 | 17.31 | 17.28 | 17.29 | 18.18 | 19.67 | 21.86 | 23.38 | 24.75 | 24.77 |
| **Number of prescriptions with a bronchodilator response per patient per year** | | | | | | | | | | |
| Total | 1.15 | 1.17 | 1.17 | 1.17 | 1.17 | 1.17 | 1.18 | 1.20 | 1.21 | 1.22 |
| Non-severe | 1.12 | 1.13 | 1.13 | 1.14 | 1.15 | 1.15 | 1.16 | 1.18 | 1.19 | 1.20 |
| Severe | 1.35 | 1.39 | 1.39 | 1.37 | 1.32 | 1.35 | 1.36 | 1.42 | 1.45 | 1.51 |
| **Skin prick test**  **Percentage of patients with a skin prick test prescription** | | | | | | | | | | |
| Total (%) | 1.81 | 1.57 | 1.43 | 1.37 | 1.30 | 1.22 | 1.12 | 1.14 | 1.06 | 0.96 |
| Non-severe (%) | 1.70 | 1.50 | 1.37 | 1.33 | 1.27 | 1.19 | 1.10 | 1.12 | 1.05 | 0.94 |
| Severe (%) | 5.16 | 3.57 | 3.39 | 2.92 | 2.71 | 2.52 | 2.33 | 2.36 | 2.01 | 1.95 |
| **Number of prescriptions with a skin prick test per patient per year** | | | | | | | | | | |
| Total | 1.01 | 1.01 | 1.01 | 1.01 | 1.01 | 1.01 | 1.01 | 1.01 | 1.01 | 1.01 |
| Non-severe | 1.01 | 1.01 | 1.01 | 1.01 | 1.01 | 1.01 | 1.01 | 1.01 | 1.01 | 1.01 |
| Severe | 1.02 | 1.02 | 1.02 | 1.02 | 1.02 | 1.01 | 1.02 | 1.03 | 1.01 | 1.01 |
| **Chest radiography**  **Percentage of patients with a chest radiograph prescription** | | | | | | | | | | |
| Total (%) | 28.67 | 26.32 | 23.67 | 23.05 | 22.10 | 22.12 | 20.44 | 19.85 | 19.99 | 21.62 |
| Non-severe (%) | 27.78 | 25.44 | 22.77 | 22.17 | 21.23 | 21.43 | 19.80 | 19.19 | 19.39 | 21.05 |
| Severe (%) | 55.79 | 54.84 | 54.59 | 55.32 | 56.44 | 57.85 | 58.33 | 58.21 | 58.19 | 57.51 |
| **Number of prescriptions with chest radiographs per patient per year** | | | | | | | | | | |
| Total | 1.42 | 1.44 | 1.47 | 1.49 | 1.52 | 1.49 | 1.51 | 1.51 | 1.50 | 1.51 |
| Non-severe | 1.38 | 1.40 | 1.42 | 1.44 | 1.47 | 1.45 | 1.46 | 1.46 | 1.45 | 1.47 |
| Severe | 2.03 | 2.05 | 2.13 | 2.22 | 2.32 | 2.36 | 2.49 | 2.47 | 2.49 | 2.50 |
| **Chest computed tomography (CT)**  **Percentage of patients with a chest CT prescription** | | | | | | | | | | |
| Total (%) | 1.24 | 1.36 | 2.00 | 2.21 | 2.42 | 2.53 | 2.42 | 2.56 | 2.56 | 2.92 |
| Non-severe (%) | 1.11 | 1.22 | 1.76 | 1.95 | 2.15 | 2.29 | 2.20 | 2.32 | 2.34 | 2.69 |
| Severe (%) | 5.25 | 6.05 | 10.13 | 11.40 | 13.20 | 14.99 | 15.58 | 16.39 | 16.42 | 17.28 |
| **Number of prescriptions with a chest CT per patient per year** | | | | | | | | | | |
| Total | 1.08 | 1.08 | 1.12 | 1.14 | 1.17 | 1.66 | 1.16 | 1.17 | 1.17 | 1.18 |
| Non-severe | 1.07 | 1.07 | 1.10 | 1.12 | 1.14 | 1.63 | 1.14 | 1.15 | 1.15 | 1.16 |
| Severe | 1.12 | 1.14 | 1.22 | 1.26 | 1.30 | 1.92 | 1.32 | 1.35 | 1.35 | 1.37 |
| **Para-nasal sinus (PNS) radiography**  **Percentage of patients with a PNS radiograph prescription** | | | | | | | | | | |
| Total (%) | 4.27 | 3.82 | 3.86 | 4.02 | 4.01 | 4.03 | 4.06 | 4.40 | 4.66 | 4.86 |
| Non-severe (%) | 4.02 | 3.62 | 3.66 | 3.86 | 3.86 | 3.90 | 3.94 | 4.27 | 4.54 | 4.74 |
| Severe (%) | 12.01 | 10.40 | 10.68 | 9.83 | 9.98 | 10.69 | 11.18 | 12.04 | 12.50 | 12.41 |
| **Number of prescriptions with PNS radiographs per patient per year** | | | | | | | | | | |
| Total | 1.11 | 1.12 | 1.13 | 1.15 | 1.15 | 1.14 | 1.17 | 1.19 | 1.19 | 1.18 |
| Non-severe | 1.10 | 1.11 | 1.12 | 1.13 | 1.14 | 1.14 | 1.16 | 1.18 | 1.17 | 1.17 |
| Severe | 1.22 | 1.23 | 1.24 | 1.28 | 1.28 | 1.31 | 1.38 | 1.44 | 1.47 | 1.52 |
| **Multiple allergen simultaneous test (MAST)**  **Percentage of patients with a MAST prescription** | | | | | | | | | | |
| Total (%) | 0.67 | 0.69 | 0.68 | 0.83 | 0.86 | 0.93 | 0.98 | 1.13 | 1.20 | 1.39 |
| Non-severe (%) | 0.64 | 0.66 | 0.66 | 0.80 | 0.84 | 0.91 | 0.96 | 1.10 | 1.17 | 1.37 |
| Severe (%) | 1.65 | 1.54 | 1.57 | 1.82 | 1.74 | 1.91 | 2.10 | 2.61 | 2.80 | 2.75 |
| **Number of prescriptions with a MAST per patient per year** | | | | | | | | | | |
| Total | 1.01 | 1.01 | 1.01 | 1.02 | 1.01 | 1.01 | 1.01 | 1.01 | 1.01 | 1.02 |
| Non-severe | 1.01 | 1.01 | 1.01 | 1.01 | 1.01 | 1.01 | 1.01 | 1.01 | 1.01 | 1.01 |
| Severe | 1.02 | 1.02 | 1.04 | 1.04 | 1.03 | 1.03 | 1.03 | 1.03 | 1.05 | 1.06 |
| **Serum-specific IgE test (ImmunoCAP)**  **Percentage of patients with an ImmunoCAP prescription** | | | | | | | | | | |
| Total (%) | 0.77 | 0.71 | 0.72 | 0.72 | 0.65 | 0.57 | 0.51 | 0.56 | 0.57 | 0.59 |
| Non-severe (%) | 0.71 | 0.67 | 0.66 | 0.68 | 0.62 | 0.55 | 0.49 | 0.54 | 0.56 | 0.57 |
| Severe (%) | 2.70 | 2.21 | 2.51 | 2.16 | 1.84 | 1.77 | 1.59 | 1.58 | 1.54 | 1.53 |
| **Number of prescriptions with an ImmunoCAP per patient per year** | | | | | | | | | | |
| Total | 1.04 | 1.04 | 1.04 | 1.04 | 1.04 | 1.03 | 1.04 | 1.03 | 1.05 | 1.05 |
| Non-severe | 1.04 | 1.03 | 1.04 | 1.03 | 1.04 | 1.03 | 1.04 | 1.03 | 1.05 | 1.04 |
| Severe | 1.07 | 1.04 | 1.07 | 1.06 | 1.05 | 1.08 | 1.10 | 1.07 | 1.07 | 1.11 |
